# ANXA5: related mechanisms of osteogenesis and additional biological functions

**DOI:** 10.3389/fcell.2025.1553683

**Published:** 2025-04-24

**Authors:** Ming Jin, Jingrun Zhang, Yimeng Sun, Ge Liu, Xiaowei Wei

**Affiliations:** ^1^ Zhongshan Clinical College, Dalian University, Dalian, China; ^2^ National and Local Joint Engineering Laboratory for Orthopedic Implant Material Development, Affiliated Zhongshan Hospital of Dalian University, Dalian, China

**Keywords:** ANXA5, phosphatidylserine, bone regeneration, biological functions, signaling pathways

## Abstract

Annexin A5 (ANXA5), also known as Annexin V, is a calcium-dependent phospholipid-binding protein and has a high affinity with phosphatidylserine (PS). This characteristic facilitates its involvement in a wide range of biological functions, including vesicle transport, the formation of mineral phases in the extracellular matrix, anticoagulation and antithrombotic, the inhibition of tumor growth, and apoptosis regulation. ANXA5 plays a role in anti-inflammatory and antithrombotic properties. It also has protective effects on the nervous system. ANXA5 has been reported to facilitate osteogenic differentiation and take part in chondrocyte apoptosis and mineralization. More and more attention is paid to the potential of ANXA5 for bone defect repair. Most current studies on ANXA5 mainly concentrate on immune disorders, pregnancy disorders and serve as a biomarker for various diseases as well as apoptosis detection. However, there is still a lack of systematic studies on ANXA5 involving multiple tissues, including bone, cartilage, vessels, and nerves in the process of bone regeneration. Our study aims to summarize the biological functions in bone tissue and the related signaling pathways of ANXA5. This work provides a theoretical foundation for applying ANXA5 in clinical orthopedics in the future.

## 1 Introduction

Annexins (ANXs) comprise a multigene family consisting of approximately 500 members, which are found in vertebrates, invertebrates, fungi, plants, and protozoa. Twelve kinds of common Annexins in vertebrates belong to the Annexin A family, named ANXA1 to ANXA11 and ANXA13 (Moss and Morgan, 2004). Annexins are classified into several families: the B family (invertebrates), the C family (fungi and certain unicellular eukaryotes), the D family (plants) and the E family (protozoa) (Gerke et al., 2005; Xi et al., 2020).

**FIGURE 1:**
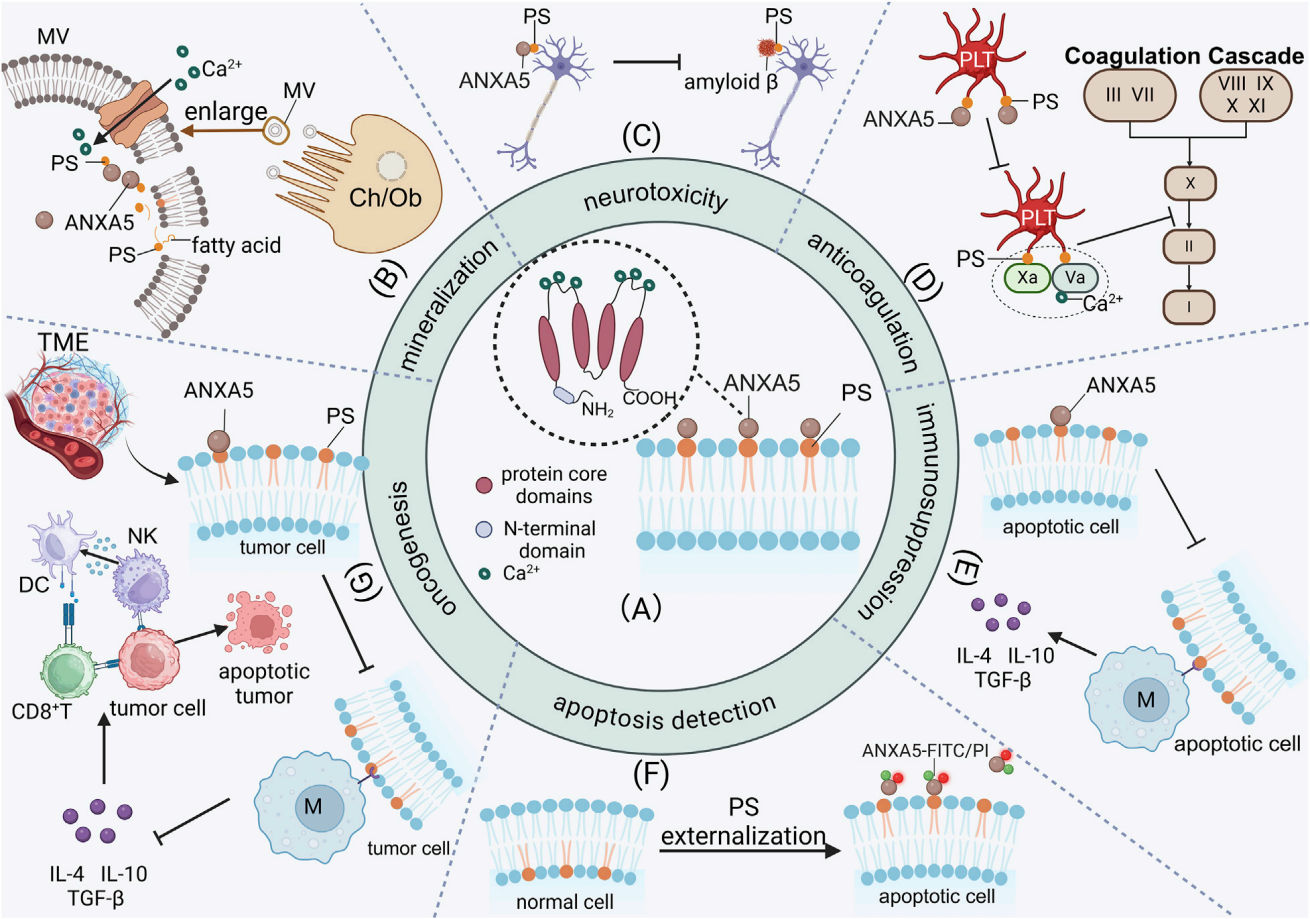
The high affinity of ANXA5 with PS facilitates its participation in a wide range of biological processes. **(A)** The ANXA5 has a conserved C-terminal core domain and an N-terminal domain. The C-terminal is a core domain composed of four repeated sequences. The C-terminal has calcium-binding motifs and mediates the binding to negatively charged phospholipids. **(B)** In the extracellular matrix of osteoblasts and chondrocytes, ANXA5 facilitates mineralization by binding to PS-rich matrix vesicle membranes, acceleratingCa^2+^ influx and subsequently promoting hydroxyapatite deposition in the extracellular matrix. **(C)** The characteristic of ANXA5 inhibits the binding between amyloid β and PS, resulting in the accumulation of neurotoxic amyloid β in the choroid plexus of Alzheimer’s disease patients. **(D)** ANXA5 binds to and interacts with activated platelets, competitively inhibiting the binding of coagulation factors (Va and Xa) to activated platelets, thus preventing the initiation of the coagulation cascade. **(E)** ANXA5 inhibits macrophages phagocytosis of apoptotic cells and promotes the release of immunosuppressive cytokines, including IL-4, IL-10, and TGF-β thereby exerting immunosuppression. **(F)** The characteristic of ANXA5 is particularly significant because of PS externalization during cell apoptosis, making ANXA5 a key agent for detecting cell apoptosis *in vivo.* **(G)** In tumor cells, ANXA5 inhibits macrophages from secreting immunosuppressive cytokines IL-4, IL-10, and TGF-β by blocking PS exposure on tumor cells. Dendritic cells regain the ability to present antigens to CD8^+^T cells, shifting the immune profile from immunosuppressive to immune-active. MV, Matrix vesicle; PS, Phosphatidylserine; PLT, Platelet; Ch, Chondrocytes; Ob, Osteoblasts; M, Macrophage; TME, Tumor microenvironment; DC, Dendritic cells; NK, Natural killer cell.

ANXA5 (Annexin A5), also known as Annexin V, is a key member of the Annexin A family. ANXA5 was first found by Bohn et al., who isolated the ANXA5 protein from the human placenta and designated it as placental protein 4 (PP4) (Inaba et al., 1984). It is also called calphobindin-I(CPB-I), lipocortin V, placental anticoagulant protein I (PAP-I), endonexin II(E-II), vascular anticoagulant-α(VAC α) and anchorin CII. ANXA5 is a single-chain protein weighing 35–36 kD (Jing, 2024), and it is encoded by a gene located on chromosomes 4q26-q28 (Tait et al., 1991). ANXA5 forms protein-protein interactions by binding to specific sites of the S100 dimer (Rescher and Gerke, 2004; Rintala-Dempsey et al., 2008). The ANXA5 has a conserved C-terminal domain and an N-terminal domain. The C-terminal is a core domain composed of four repeated sequences. The core domain contains approximately 70 amino acid residues (Gerke and Moss, 2002; Lizarbe et al., 2013; Xi et al., 2020). Each repeated sequence of the C-terminal has five α-helices, including calcium-binding motifs and mediating the binding to negatively charged phospholipids. The N-terminal domain of ANXA5 contains calcium-binding sites and phosphorylation sites (Qi et al., 2015; Woodward et al., 2022). (Figure 1A)

ANXA5 is found in different tissues. Noteworthily, ANXA5 is known for its ability to bind to phosphatidylserine (PS) on cell membranes with high affinity in a calcium-dependent manner (Boersma et al., 2005; Schutters and Reutelingsperger, 2010). This characteristic is particularly significant, making ANXA5 participate in various biological processes *in vivo*, including vesicle transport, mineralization in the extracellular matrix, neurotoxicity in Alzheimer’s disease, anticoagulation and antithrombotic, apoptosis regulation and detection, and inhibition of tumor cell growth (Krey et al., 2016). In the extracellular matrix of osteocytes and chondrocytes, ANXA5 facilitates mineralization through its binding to PS-rich matrix vesicle membranes, which accelerates Ca^2+^ influx and subsequently promotes the deposition of hydroxyapatite crystals in the extracellular matrix (Ansari et al., 2021; Wuthier and Lipscomb, 2011; Genge et al., 2008) (Figure 1B). Furthermore, the characteristic of ANXA5 for binding to PS also plays a role in neurological disorders. For example, the characteristic of ANXA5 inhibits the binding between amyloid β and PS, resulting in the accumulation of neurotoxic amyloid β in the choroid plexus of Alzheimer’s disease patients, ultimately leading to cellular death (Bartolome et al., 2020; Lee et al., 2002) (Figure 1C). Additionally, ANXA5 has an antithrombotic function in the blood vessels. ANXA5 can displace coagulation factors from procoagulant phospholipids, thereby inhibiting the coagulation cascade *in vitro* (Cederholm and Frostegård, 2007; Reddy and Rand, 2020; Ravanat et al., 1992) (Figure 1D). The disruption of the anticoagulation pathway involving ANXA5 is associated with antiphospholipid syndrome (Bogdanova et al., 2012; Mineo et al., 2023) and its pregnancy complications, including recurrent pregnancy loss (Aranda et al., 2018; Rogenhofer et al., 2018; Ang et al., 2017). ANXA5 also has anti-inflammatory properties. The binding between ANXA5 and PS effectively inhibits PS-mediated adhesion of platelets and white blood cells to the endothelium, consequently mitigating systemic inflammation (Ewing et al., 2011; Ewing et al., 2012). PS mainly exists in the inner leaflets of cells. The characteristic of ANXA5 is particularly significant because of PS externalization during cell apoptosis, making ANXA5 a key agent for detecting cell apoptosis *in vivo* (Rieger et al., 2011; Kumar et al., 2021; Krysko et al., 2004; Miyagishima et al., 2024) (Figure 1F). PS externalization serves as a primary signal for macrophages to recognize and clear apoptotic cells (Munoz et al., 2007). ANXA5 selectively binds to PS on the surface of apoptotic cells with high affinity, thereby competitively inhibiting the phagocytosis of apoptotic cells by macrophages and consequently exerting an immunosuppressive effect (Stach et al., 2000; Böttcher et al., 2006; Munoz et al., 2007) (Figure 1E). In tumor cells, ANXA5 enables dendritic cells to regain their capacity for antigen presentation to CD8^+^ T cells by blocking PS exposure on tumor cells. Furthermore, ANXA5 inhibits the secretion of immunosuppressive cytokines in macrophages, thereby shifting the immune profile from immunosuppressive to immune-active. (Yan et al., 2012; Bondanza et al., 2004; Kang et al., 2020) (Figure 1G). Through this mechanism, ANXA5 alleviates the immunosuppressive effects induced by chemotherapy and enhances the antitumor efficacy and immunogenicity of tumor antigen-specific immune responses (Bondanza et al., 2004; Chaurio et al., 2009; Frey et al., 2009; Gray et al., 2016).

Bone regeneration is a complex physiological process that involves the co-regulation of various phenomena, including endochondral ossification, osteogenic differentiation, angiogenesis, neurogenesis, and so on (Salhotra et al., 2020). Numerous studies have indicated that ANXA5 is able to promote osteoblast differentiation and prevent osteoporosis (Su et al., 2023; Genetos et al., 2014; Shimada et al., 2018). Additionally, ANXA5 facilitates the apoptosis and terminal differentiation of chondrocytes and promotes chondrocyte mineralization, indicating its significant role in bone regeneration (Shimada et al., 2018; Wang and Kirsch, 2006; Kirsch et al., 2000). Currently, there are no reviews on ANXA5 in bone tissue. Most reviews focus on cardiovascular disease (Cederholm and Frostegård, 2007), immune disorders (Bećarević, 2016; Rand et al., 2010), tumorigenesis (Woodward et al., 2022; Peng et al., 2014), pregnancy complications (Bogdanova et al., 2012; Peng et al., 2022)and the detection of apoptosis (Laufer et al., 2008). In this study, we provide a detailed description of these physiological processes and summarize the biological functions and associated signaling pathways of ANXA5 within the field of bone tissue. We aim to provide a theoretical foundation for applying ANXA5 in clinical orthopedics in the future.

## 2 The function of ANXA5 in bone tissue

### 2.1 The expression of ANXA5 in bone tissue

Suarez et al. isolated osteoblasts from the skull of newborn rats and detected the cellular protein extracts of these osteoblasts by Western blotting. They found that ANXA5 was expressed in the osteoblast cell lineage (Suarez et al., 1993). Brachvogel et al. analyzed ANXA5 in frozen sections of mouse embryos through immunohistochemistry, discovering that ANXA5 was present in frozen sections of the developing lumbar arch. The ANXA5 gene plays a role in the cellular lineage of skeletal system development, and the researchers believe it may represent a novel marker characterizing involved in this process (Brachvogel et al., 2001). Su et al. detected the expression of ANXA5 in bone tissue *in vivo* and primary osteoblasts *in vitro* from both sham and osteoporotic mice by Western blotting (Su et al., 2023). Mohiti et al. obtained bone tissue from human knee joints and then isolated osteoblasts, confirming the presence of ANXA5 in these cells through Western blotting. They determined that the cellular content of ANXA5 was found to be 0.18% ± 0.010% (n = 9) of total cell protein in primary cultures of osteoblasts using quantitative immunoblotting. Additionally, the localization of ANXA5 in MG-63 osteosarcoma cells was determined using immunofluorescence microscopy, which revealed that ANXA5 was always strongly present in the nucleus with additional cytoplasmic staining (Mohiti et al., 1995).

### 2.2 ANXA5 promotes the proliferation and differentiation of osteogenic cells

After knocking down the ANXA5 gene in preosteoblast MC3T3 cells, Genetos et al. observed a reduction in cell proliferation, as measured by Calcein-AM and Alamar Blue staining. When MC3T3 cells were cultured under osteogenic differentiation-inducing conditions, ANXA5 revealed maximal expression at 14 days. These findings suggest that ANXA5 can influence bone formation via the regulation of osteoprogenitor proliferation and differentiation in addition to the function in matrix vesicles (MVs) (Genetos et al., 2014). Shimada et al. reported that after knocking down the ANXA5 gene in primary cultures of osteoblasts *in vitro*, subsequent qPCR analysis resulted in decreased Runx2 and osteopontin expression. This suggests that ANXA5 plays a role in promoting osteoblast differentiation (Shimada et al., 2018). Furthermore, Su et al. found that ANXA5 expression was significantly downregulated in bone tissue and isolated osteoblasts of osteoporosis mice compared to those of the sham mice. After transfecting the shANXA5 plasmid into the preosteoblastic cell line MC3T3, Western blot analysis indicated a significant reduction in osteogenic differentiation-related markers (Su et al., 2023).

However, further research conducted by Brachvogel et al. indicates that ANXA5 is not essential for bone development. In ANXA5-deficient mouse mutants, no serious defects related to the ossification process were observed. X-ray analyses of the skeleton from 6-month-old mice revealed no significant differences in size or the density of the bone. Additionally, histological analysis of the tibia from newborn mice displayed no overt changes in the organization of the growth plate in the absence of ANXA5. These findings demonstrate that mice lacking ANXA5 can develop normally and reveal no significant alterations in the biochemical parameters characteristic of metabolic or functional defects. This may be due to a compensatory effect of other members from the annexin family arising from the high functional and structural similarity (Brachvogel et al., 2003).

### 2.3 ANXA5 enhances the mineralization process in osteoblasts

Su et al. conducted a study in which they transfected the shANXA5 plasmid into the preosteoblast cell line MC3T3. The results from ALP staining and alizarin red staining showed that shANXA5 decreased the number of ALP-positive cells and inhibited the formation of mineralized nodules. Conversely, in cells that overexpressed ANXA5, these results were significantly enhanced, confirming that ANXA5 mediates the mineralization of the precursor osteoblast lineage (Su et al., 2023). Additionally, when Genetos et al. applied ANXA2 siRNA and ANXA5 siRNA to preosteoblast MC3T3 cells, they observed a decrease in osteogenic marker ALP staining and a significant reduction in mineralized nodule formation after knockdown. This further supports that ANXA5 plays a significant role in promoting osteogenic differentiation (Genetos et al., 2014).

**FIGURE 2:**
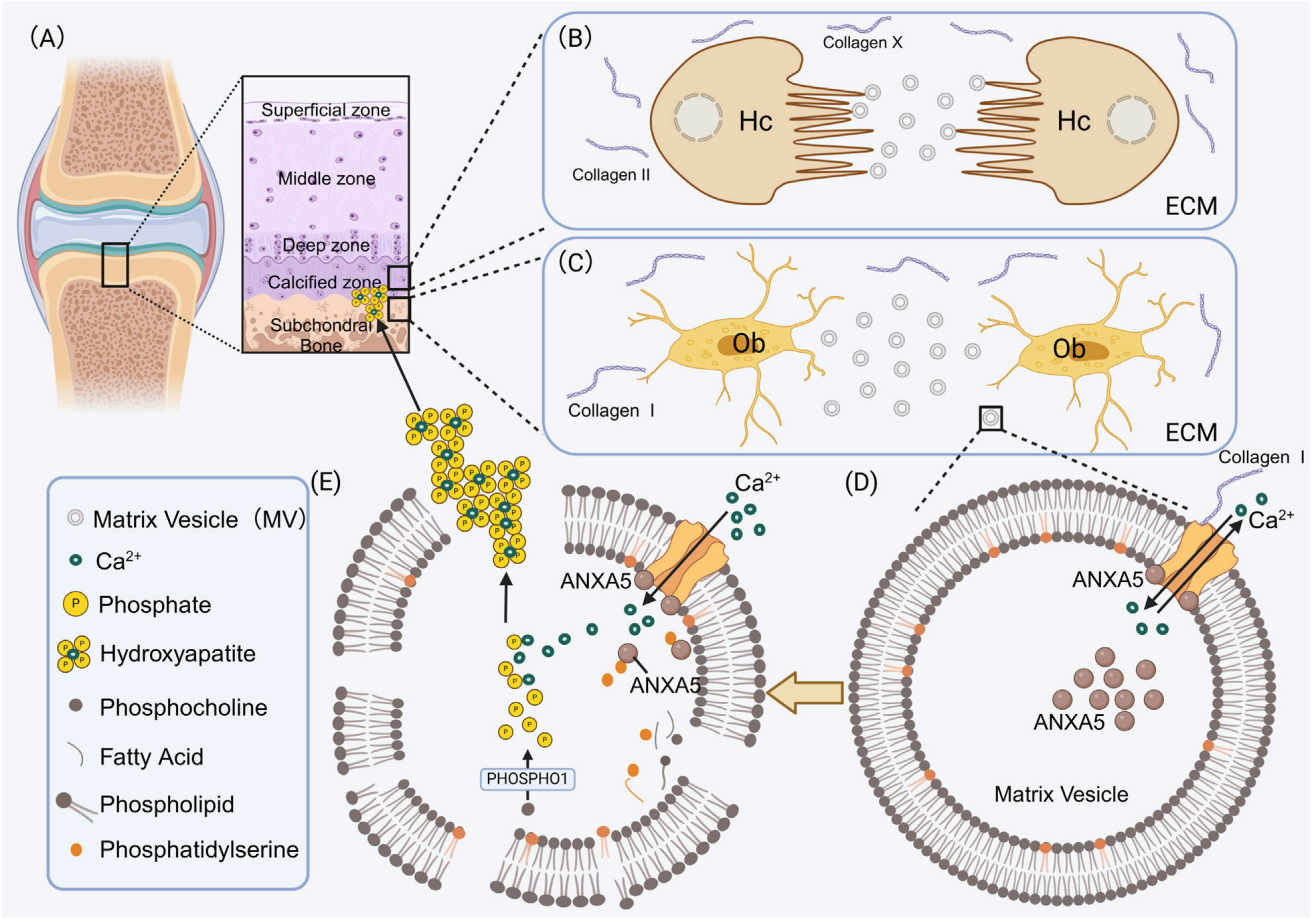
ANXA5 participates in the mineralization process of the extracellular matrix in osteoblasts and chondrocytes. **(A)** Hypertrophic chondrocytes form mineralized cartilage tissue in the calcified zone; osteoblasts form mineralized bone tissue in the subchondral bone. **(B)** Type II and Type X collagens constitute the primary components of the extracellular matrix in hypertrophic chondrocytes. **(C)** Type I collagens constitute the primary components of the extracellular matrix in osteoblasts. MVs sprout from the surface of osteoblasts and subsequently enter the extracellular matrix. **(D)** In the MVs secreted by osteoblasts or hypertrophic chondrocytes, Ca^2+^ transport ANXA5-mediated maintains MVs homeostasis. For instance, in MVs secreted by osteoblasts, type I collagens bind to ANXA5 on the surface of MVs, thereby accelerating Ca^2+^ influx. ANXA5 serves not only as a transmembrane calcium channel but also as a calcium-binding protein that accumulates within the MVs. **(E)** The membranes of MVs are abundant in PS. In the presence of calcium, ANXA5 binds to PS, thereby accelerating the Ca^2+^ influx. Phosphocholines release phosphates through PHOSPHO1, which combines with calcium ions to form hydroxyapatite crystals inside the MVs. Hydroxyapatite crystals are deposited in the extracellular matrix to complete the mineralization of cartilage. Hc, Hypertrophic chondrocytes; Ob, Osteoblasts; ECM, Extracellular matrix; PHOSPHO1, Phosphoethanolamine/Phosphocholine phosphatase1; Collagen I, type I collagen; Collagen II, type II collagen.

Su also found that ANXA5 is highly expressed in osteoblast adhesion MVs (Su et al., 2023). It is widely known that type I collagen is the main organic extracellular matrix component in osteoblasts (Kim and Kirsch, 2008) (Figure 2C). In MC3T3 cells with ANXA5 knockdown, there was a decrease in the number of MVs attaching to the cellular matrix, which suggests that ANXA5 may regulate the interaction between MVs and the extracellular matrix. In both *in vivo* bone tissue and *in vitro* osteoblasts, immunofluorescence double staining revealed colocalization between ANXA5 and type I collagen. Subsequent co-immunoprecipitation experiments demonstrated the direct binding between MVs and type I collagen. These results further confirm that ANXA5, located on the MVs membrane, can directly attach to type I collagen. Therefore, it is suggested that ANXA5 may play a protective role against bone loss by promoting MVs adhesion to the extracellular matrix through its interaction with type I collagen (Su et al., 2023) (Figure 2D).

## 3 The function of ANXA5 in cartilage tissue

### 3.1 The expression of ANXA5 in cartilage tissue

ANXA5 is essential for the normal proliferation and hypertrophy of chondrocytes. Castagnola et al. demonstrated that ANXA5 mRNA reaches its maximum level in hypertrophic stage II chondrocytes (Castagnola and Cancedda, 1991). Rahman and Giambanco showed that ANXA5 expression is closely linked to the differentiation of chondrocytes and skeletal muscle cells during limb development (Rahman et al., 1997; Giambanco et al., 1991). Shimada et al. examined the entire tibia and femur of ANXA5^+/−^mice. They found that ANXA5 is expressed at the tibial attachment site, the periosteum, the articular cartilage surface, and the growth plate cartilage (Shimada et al., 2018).

### 3.2 ANXA5 enhances chondrocyte mineralization

#### 3.2.1 ANXA5 interacts with type II and type X collagen, accelerating the Ca^2+^ influx and promoting chondrocyte mineralization

Unlike osteoblasts, ANXA5 on the surface of MVs secreted by chondrocytes plays a crucial role in chondrocyte mineralization (Figure 2A). This function is achieved through its interaction with extracellular matrix components, specifically type II collagen and type X collagen (Wuthier and Lipscomb, 2011). (Figure 2B) Several researchers have shown that ANXA5 is involved in the interaction between chondrocytes and extracellular collagen, contributing to the mineral deposition process in primary cultures of chicken growth plate chondrocytes grown in ascorbate-containing media (Mollenhauer et al., 1984; Castagnola and Cancedda, 1991; Pfäffle et al., 1988; Mebarek et al., 2023). Kirsch et al. provided evidence using slot blot assays that ANXA5 not only binds to native type II and X collagen but also to chondrocalcin, the C-terminal extension of type II collagen in a calcium-independent manner (Kirsch and Pfäffle, 1992). Bolean et al. found that ANXA5-lipoprotein complexes have the highest affinity for type II collagen deposited during chondrocyte mineralization in articular cartilage (Bolean et al., 2020). King et al. discovered that *in vitro* cultured Swarm rat chondrosarcoma cells lack the ability to bind significant amounts of type II collagen to their surfaces, as compared to normal rat chondrocytes, which correlates with a deficiency of ANXA5 on these cell surfaces (King et al., 1997). Lucic et al. discovered that the N-terminal peptide of type II collagen binds to ANXA5. The binding of C-terminal peptides and triple helical peptide to the chondrocyte surface may occur through other collagen receptors, such as integrins or cell-associated matrix proteins (Lucic et al., 2003). Furthermore, von der Mark K et al. demonstrated that during matrix vesicle-initiated cartilage mineralization, the binding of ANXA5 to collagen significantly facilitates calcium influx into MVs (von der Mark and Mollenhauer, 1997).

The N-terminal domain of ANXA5 has been shown to contain calcium-binding sites that facilitate the influx of calcium (Kim and Kirsch, 2008). ANXA5, which is expressed on the surface of MVs secreted by chondrocytes, interacts with type II and type X collagen. This interaction mediates the flow of Ca^2+^ into the MVs released by hypertrophic cartilage in the growth plate (Boyan et al., 2022; Anderson, 2003). Phosphocholines release phosphates through PHOSPHO1 (Phosphoethanolamine/Phosphocholine phosphatase), which combines with calcium ions to form Hydroxyapatite crystals inside of the MV. Hydroxyapatite crystals are deposited in the extracellular matrix to complete the mineralization of cartilage (Bolean et al., 2017; Wuthier and Lipscomb, 2011; Chaudhary et al., 2016; Millán, 2013) (Figure 2E). Additionally, due to the close association of matrix vesicles with the extracellular matrix rich in collagen and proteoglycans, Genge BR et al. speculate that this MV protein may be a stretch-activated ion channel component that enhances Ca^2+^ uptake during mechanical stress (Genge et al., 1992).

#### 3.2.2 ANXA5 interacts with PS in MVs to accelerate the Ca^2+^ influx and enhance the mineralization of chondrocytes

ANXA5 is known for its high-affinity binding to PS on cell membranes, a process that depends on calcium concentrations (von der Mark and Mollenhauer, 1997). Research by Köhler et al. has shown that the binding affinity of recombinant ANXA5 proteins to PS-rich MVs is stronger at low pH compared to neutral pH (Köhler et al., 1997). Furthermore, studies by Genge BR and Kirsch T et al. have demonstrated that the membranes of MVs *in vivo* are rich in PS, which binds to ANXA5. This interaction enhances calcium (Ca^2+^) influx and promotes intracavicular crystal growth, thereby playing a critical role in the mineralization of the cartilage matrix (Genge et al., 2007; Kirsch et al., 1997).

### 3.3 ANXA5 promotes apoptosis in cartilage

ANXA5 plays a crucial role in promoting chondrocyte mineralization, which ultimately leads to apoptosis. A key characteristic of osteoarthritis is the eventual apoptosis of chondrocytes following mineralization (Wang et al., 2023a). Research by Mollenhauer has demonstrated that ANXA5 is significantly upregulated in the cartilage of patients with osteoarthritis. The expression and distribution of ANXA5 could serve as a histological marker for metabolic alterations and changes in cell phenotype associated with osteoarthritis (Mollenhauer et al., 1999). Shimada and colleagues have shown that ANXA5 inhibits the proliferation of fibrocartilage and prevents the excessive growth of bone ends (Shimada et al., 2018). Wang et al. discovered that ANXA5 alters Ca^2+^ homeostasis in growth plate chondrocytes, thereby regulating terminal differentiation and mineralization events (Wang et al., 2005). Additionally, they also found that the binding of ANXA5 to active protein kinase Cα (PKCα) stimulates apoptosis in growth plate chondrocytes. Meanwhile, the interaction of ANXA5 with β5 integrin regulates these processes, ultimately leading to apoptosis (Wang and Kirsch, 2006). Furthermore, research by Kirsch et al. found that human osteoarthritic chondrocytes adjacent to the joint space undergo terminal differentiation and release MVs containing ANXA5, which initiate mineral formation and eventually die by apoptosis (Kirsch et al., 2000).

## 4 The function of ANXA5 in vessels

### 4.1 The expression of ANXA5 in vessels

Brachvogel et al. used mice with the ANXA5-lacZ fusion gene to investigate the expression of ANXA5 in mouse blood vessels. Their initial findings revealed that the fusion gene is expressed in cells associated with the embryonic vascular network (Brachvogel et al., 2001). Further research demonstrated that, following X-gal staining of embryonic sections, ANXA5 expression is limited to the primary capillary plexus, the dorsal aorta and extraembryonic tissue during early embryonic development. Perivascular cells (PVCs) are crucial for proper vascular development and play a significant role in maintaining both the structural integrity and contractility of vessels (Brachvogel et al., 2005).

### 4.2 ANXA5 reduces inflammation in endothelial

Ewing et al. conducted a study demonstrating that ANXA5 reduces local vascular and systemic inflammation and vascular remodeling and improves vascular function, indicating that it has a therapeutic potential against atherosclerotic cardiovascular diseases (Ewing et al., 2011). Tschirhart et al. found that recombinant human ANXA5 protein, by binding to PS, can inhibit endothelial inflammation induced by microvesicles in septic patients (Tschirhart et al., 2023). Additionally, *in vitro* experiments by Burgmaier et al. revealed that ANXA5 significantly inhibits the capture, rolling, adhesion, and migration of peripheral blood mononuclear cells on TNF-α-activated endothelial cells within inflammatory lesions. The research team also observed that short-term treatment with ANXA5 can decrease inflammation in plaque lesions of atherosclerotic mice by interfering with the recruitment and activation of monocytes at sites of inflammation (Burgmaier et al., 2014).

### 4.3 ANXA5 suppresses the apoptosis of endothelial cells

Liu et al. demonstrated that knocking out the ANXA5 gene in human umbilical vein endothelial cells (HUVECs) led to a decline in cell viability. Flow cytometry analysis showed that the knockout of ANXA5 promotes apoptosis in HUVECs (Liu et al., 2024). Additionally, it has been reported that anti-ANXA5 antibodies can induce apoptosis in vascular endothelial cells. The clinical study by Habeeb et al. indicated that patients with systemic sclerosis (SSc) have anti-ANXA5 antibodies in their serum and that higher antibody titers are associated with more severe vascular damage (Habeeb et al., 2010; Sugiura and Muro, 1999). Furthermore, Tripathy et al. observed a significant increase in anti-ANXA5 antibody levels in patients with Takayasu’s arteritis (TA), and a corresponding increase in the number of anti-endothelial cell antibodies (AECA) positively correlated. Anti-ANXA5 antibodies were also positively correlated with disease activity, suggesting they play a pathogenic role in the disease (Tripathy et al., 2003).

### 4.4 ANXA5 promotes angiogenesis and vascular differentiation

Brachvogel et al. isolated perivascular cells (PVC) from ANXA 5-LacZ + mice that specifically expressed ANXA 5 and discovered that they possessed the ability to differentiate into various mesenchymal lineages (Brachvogel et al., 2005). Subsequently, they added growth factors, namely, vascular endothelial growth factor (VEGF) and platelet-derived growth factor (PDGF), to the cell culture medium of PVC from ANXA 5-LacZ + mice. As a consequence, characteristic basement membrane proteins were detected in PVC, which are indicators of mature vascular structures (Brachvogel et al., 2007). Sun et al. discovered that the knockdown of ANXA5 was positively associated with the decrease in the levels of CD34 and VEGF-3, two indicators of angiogenesis, in mice transplanted with liver cancer cells where ANXA5 was knocked down, thereby inhibiting the progression and metastasis of liver cancer *in vivo* (Sun et al., 2018). The study conducted by Zheng et al. demonstrated that ANXA5 expression is positively correlated with the total vessel length per field in patient liver cancer tissue. Co-culturing human umbilical HUVEC with liver cancer cells HuH-7 of different ANXA5 expression levels revealed that overexpression of ANXA 5 in liver cancer cells enhances the tubulogenic ability of endothelial cells. Conversely, co-culturing with ANX5-knockout liver cancer cells reduced the tubulogenic ability of endothelial cells.

### 4.5 The anticoagulant and antithrombotic functions of ANXA5

The exposure of PS is crucial for the binding and activity of the prothrombinase complex on activated platelets (Thiagarajan and Tait, 1990; Sang et al., 2021; Zhao et al., 2016). ANXA5, which has a high affinity with PS, can bind to activated platelets and interact with them in a calcium-dependent manner (Connor et al., 2010; Thiagarajan and Tait, 1991). This binding competitively inhibits the attachment of coagulation factors to activated platelets, thereby reducing coagulation. As a result, ANXA5 is recognized as a highly effective anticoagulant protein. In a study by Van Ryn-McKenna et al., heparin and ANXA5 were injected into the injured carotid vein of a denuded rabbit. They discovered that ANXA5 prevented the formation of the prothrombinase complex and significantly decreased thrombus formation (Van Ryn-McKenna et al., 1993). Additionally, research conducted by Li et al. found that ANXA5 can inhibit the expression and activity of tissue factor (TF) and its release induced by homocysteine (Hcy) in vascular smooth muscle cells (VSMCs) (Li et al., 2009).

Bone regeneration is also a critical aspect of vascular development. Kusumbe and his colleagues have demonstrated that the blood vessels in the bone comprise endothelial cells that specifically facilitate bone maturation and regeneration (Kusumbe et al., 2014). While numerous studies have explored the role of ANXA5 in vascular endothelium, and several ANXA5-based drugs have been developed for anticoagulation and antithrombosis—such as recombinant ANXA5 (rANV) (van Heerde et al., 1994; Thiagarajan and Benedict, 1997), ANXA5 derivative (AND) (Ju et al., 2004; Huang et al., 2006), and recombinant ANXA5 anticoagulation fusion protein (Quan et al., 2022). However, the function of ANXA5 in angiogenesis during bone formation has not been investigated. Therefore, research in this aspect might be one of the new directions in the field of bone regeneration.

## 5 The function of ANXA5 in the nervous system

### 5.1 The expression of ANXA5 in nerve cells

Giambanco et al. investigated the cellular distribution of ANXA5 in rat tissue utilizing immunohistochemistry, revealing a pronounced positivity in glial cells within both the cerebellum and optic nerve (Giambanco et al., 1991). Additionally, Gotow et al. examined the central nervous tissue of rats using biochemical and morphological techniques. Their findings from immunoblotting and immunoelectron microscopy revealed that ANXA5 is present in neurons, with a focus on axonal terminals and synaptic vesicles. The immunoreactivity for ANXA5 is primarily localized around the cell bodies and dendrites of neurons, as observed through fluorescence and confocal laser scanning microscopy (Gotow et al., 1996).

### 5.2 The protective function of ANXA5 for neurons

Neurons are present in both the central nervous system and the peripheral nervous system and constitute the building blocks of the functional units of the nervous system. Takei N et al.'s study added recombinant human ANXA5 to embryonic rat neurons. As the amount of recombinant human ANXA5 increased, the neuron survival rate rose, reaching saturation at 30 ng/mL, demonstrating nutritional activity on neurons. Moreover, the addition of an anti-ANXA5 antibody completely inhibited this neural nutritional effect, suggesting that ANXA5 enhances neuron survival *in vitro* and functions as a paracrine neural nutrient factor in the central nervous system (Takei et al., 1994). The study revealed that in traumatic brain injury (TBI) by Gao et al., the number of apoptotic neurons in the TBI + ANXA5 group was significantly lower than that in the TBI group, indicating that ANXA5 can reduce neuronal apoptosis and exert a neuroprotective role after TBI. Furthermore, they confirmed that ANXA5 alleviates neural inflammation, oxidative stress, and iron-dependent apoptosis by regulating the NF-kB/HMGB1 pathway and the Nrf2/HO-1 antioxidant system (Gao et al., 2023). Current reports focus on neurons in the central nervous system, but there is a lack of studies regarding its role in the peripheral nervous system or its role in bone formation.

## 6 Potential role of ANXA5 in clinical disease treatment

### 6.1 Potential role of ANXA5 in bone-related disease treatment

In articles related to the use of ANXA5 for treating bone-related diseases, it is currently in the animal experimental stage, with no reports yet on its application in the clinical stage. Studies on osteoporotic mice have shown that ANXA5 treatment can relieve bone loss caused by osteoporosis, offering a novel strategy for therapeutic intervention for bone loss (Su et al., 2023). Experiments by Zhuoxuan Jia et al. demonstrated that in osteoarthritis rats induced with monosodium iodoacetate, treatment with ANXA5 effectively reduced pain symptoms and inhibited inflammation. These findings suggest new directions for treating osteoarthritis (Jia et al., 2024).

### 6.2 Potential role of ANXA5 in other diseases treatment

ANXA5 has been reported in multiple clinical trials, primarily focusing on thrombotic diseases (Quan et al., 2022), retinal vein occlusion (Wautier et al., 2011), sepsis and COVID-19 treatment (Martin et al., 2023; Mui et al., 2021). It also exhibits significant clinical potential in targeted drug delivery (Kang et al., 2020), immunotherapy in tumors (Woodward et al., 2022), treatment with systemic lupus erythematosus (Cederholm and Frostegård, 2005), atherosclerosis (Cederholm and Frostegård, 2007) and other diseases. Furthermore, ANXA5 levels may serve as a potential biomarker for preventing asthma (Lee et al., 2018), neurodegenerative disorders (Yamaguchi et al., 2010), intrauterine growth restriction and preeclampsia (Peng et al., 2022). Additionally, its levels could predict mortality in heart failure patients (Schurgers et al., 2016), evaluate lymph node metastasis and tumor grading in colon cancer patients (Sun et al., 2017) and evaluate prognosis evaluation in oral squamous cell carcinoma (Zhou et al., 2024).

## 7 Mechanisms through which ANXA5 exerts its biological functions

**FIGURE 3:**
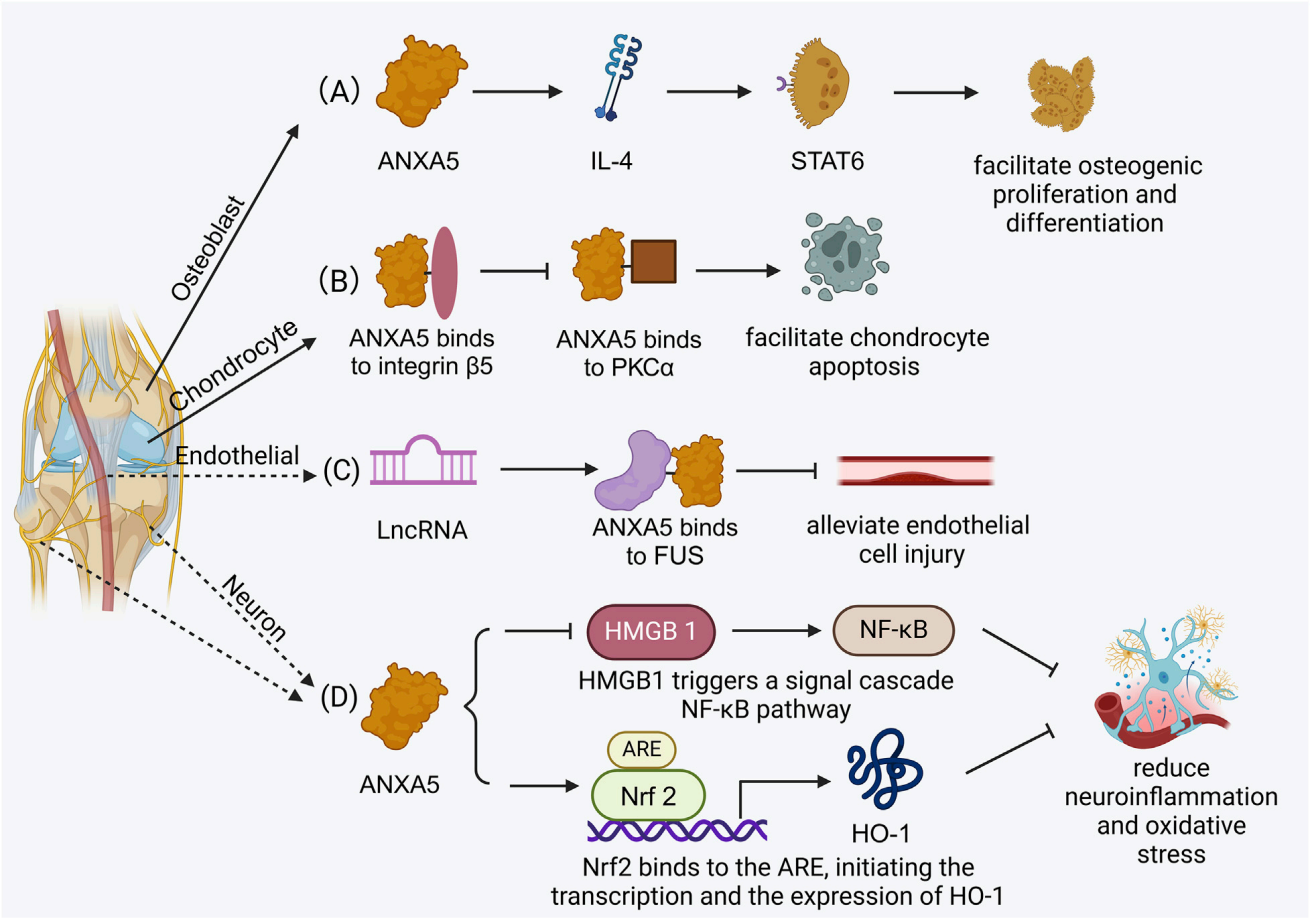
The related biological functions in bone tissue. **(A)** ANXA5 facilitates osteogenic differentiation via the STAT6 signaling pathway. **(B)** ANXA5 facilitates chondrocyte apoptosis by integrin β5/ANXA5/PKCβ signaling pathway. **(C)** ANXA5 alleviates endothelial cell injury and atherosclerosis progression through FUS/ANXA5 signaling pathway. **(D)** ANXA5 reduces injury-induced neuroinflammation and oxidative stress via NF-ĸB/HMGB1 signaling pathway and Nrf2/HO-1 signaling pathway.

ANXA5 not only promotes osteogenic differentiation, prevents the occurrence of osteoporosis, and facilitates chondrocyte mineralization and apoptosis but also enhances angiogenesis in the vascular endothelium and protects nerve cells. These critical physiological processes are integral to both bone formation and bone repair, as illustrated in Figure 3. ANXA5 also fulfills diverse functions in other normal cells, including alleviating brain injury and intestinal injury, regulating the production of testosterone and the proliferation of interstitial cells in the testis. In tumor cells, ANXA5 enhances the invasive ability of hepatocellular carcinoma cells and inhibits the expression of cyclooxygenase in prostate cancer cells. A summary of the more relevant functions and corresponding signaling pathways (or related molecular mechanism) of ANXA5 in normal tissue and tumor cells is presented below, followed by a detailed account (Table 1).

**TABLE 1:** Biological functions and related signaling pathways (or related molecular mechanism) involved in ANXA5.

Cell or tissue	Biological function	Related signal pathway (or related molecular mechanism)	Reference
Preosteoblast	ANXA5 promotes the proliferation and osteogenic differentiation of MC3T3 cells	STAT6 signaling pathway	Genetos et al. (2014)
Bone	ANXA5 derived from matrix vesicles of primary osteoblast protects against osteoporotic bone loss via mineralization	Expression of autophagy markers ATG5, ATG7, Beclin1, LC3-I and LC3-II	Su et al. (2023)
Chondrocyte	ANXA5 facilitates primary chondrocyte apoptosis	Integrin β5/ANXA5/PKCβ signaling pathway	Wang and Kirsch (2006)
Cartilage	ANXA5 prevents cartilage overgrowth at the enthesis	ANXA5 increases pyrophosphate levels by downregulating ALP and upregulating ANK and ENPP1	Shimada et al. (2018)
Macrophage	ANXA5 inhibits the polarization of macrophage to the M1 macrophage	The TLR pathway and its downstream signaling mechanisms	Jia et al. (2024)
Endothelial cells	ANXA5 alleviates HUVECs endothelial cells injury and atherosclerosis progression	lncRNAMIR4697HG/FUS/ANXA5 signaling pathway	Liu et al. (2024)
Vessels	ANXA5 promotes intravascular anticoagulant and antithromboti	ANXA5 competitively inhibits the binding of coagulation factors (Xa and Va) to PS on activated platelets	Thiagarajan and Tait, (1991)
Brain	ANXA5 ameliorates traumatic brain injury-induced neuroinflammation and neuronal ferroptosis pathways	NF-ĸB/HMGB1 and Nrf2/HO-1 signaling pathway	Gao et al. (2023), Zhang et al. (2022)
Human embryonic kidney 293T cells	ANXA5 modulates the immune response of 293T cells to IFN-γ	Jak-Stat1 signaling pathway	Leon et al. (2006)
Interstitial cells of the testis	ANXA5 induces the proliferation of TM3 Leydig cells	Ect2/RhoA/ROCK signaling pathway	Jing et al. (2015)
Testis support cells of the testis	ANXA5 protects TM4 support cells from DBP(Di-N-butylphthalate)-induced oxidative stress	ERK/Nrf2 and Nrf2/HO-1 signaling pathway	Zhang et al. (2019), Tang et al. (2020)
Prostate cancer cells	ANXA5 suppresses the proliferation of PC3 prostate cancer cells	PKC-ζ/NF-κB signaling pathway	Baek et al. (2017)
Cervical cancer cells	ANXA5 inhibits the proliferation and metastasis of HELA cervical cancer cells	PI3K/Akt signaling pathway	Wang et al. (2023b)
Liver cancer cells	ANXA5 positively regulates the proliferation, migration, invasion and *in situ* lymph node adhesion of HCA-F liver cancer cells	MEK-ERK and ERK2/c-Jun signaling pathway	Sun et al. (2018), Sun et al. (2016)
Diffuse large B-cell lymphoma cell	ANXA5 inhibits Toledo diffuse large B-cell lymphoma cell invasion and chemoresistance	PI3K/Akt signaling pathway	Wang et al. (2014)

## 8 Conclusion and perspectives

ANXA5 is expressed in bone, cartilage, vessels, and nerves. ANXA5 facilitates osteoblast differentiation. It can also enhance bone mineralization through the interaction between MVs and type I collagen, ultimately preventing the occurrence of osteoporosis. In chondrocytes, ANXA5 interacts with collagen II and collagen X via MVs to promote chondrocyte mineralization and result in chondrocyte apoptosis. Additionally, ANXA5 inhibits the apoptosis of vascular endothelial cells, and its antibody expression is associated with multiple immune system cardiovascular diseases. ANXA5 also stimulates vascular differentiation and angiogenesis. Besides, ANXA5 promotes intravascular anticoagulant and antithrombotic effects by preventing the binding of blood clotting factors to platelets. Hence, a variety of antithrombotic preparations based on ANXA5 have been developed. Furthermore, ANXA5 has a protective effect on neurons, demonstrating the properties of neurotrophic factors.

Osteogenesis represents a series of complex biological functions, such as bone and cartilage formation and revascularization, as well as the development, maintenance, and regeneration of nerves. The majority of current studies on ANXA5 mainly concentrate on immune disorders, pregnancy disorders, and apoptosis detection. Based on the relevant literature cited in this paper, future research should focus on angiogenesis and neurogenesis in the domain of bone tissue, which are crucial directions for further exploration of bone formation and the development of bone tissue engineering scaffolds.
